# Detection and characterization of circulating tumor cells in blood of primary breast cancer patients by RT-PCR and comparison to status of bone marrow disseminated cells

**DOI:** 10.1186/bcr2349

**Published:** 2009-08-10

**Authors:** Tanja Fehm, Oliver Hoffmann, Bahriye Aktas, Sven Becker, Erich F Solomayer, Diethelm Wallwiener, Rainer Kimmig, Sabine Kasimir-Bauer

**Affiliations:** 1 Department of Gynecology and Obstetrics, Calwer Straße 7, University Hospital of Tuebingen, D-72076 Tuebingen, Germany; 2 Department of Gynecology and Obstetrics, Hufelandstraße 55, University Hospital of Essen, D-45122 Essen, Germany

## Abstract

**Introduction:**

The role of circulating tumor cells (CTCs) in blood of primary breast cancer patients is still under investigation. We evaluated the incidence of CTCs in blood, we evaluated the correlation between CTCs and disseminated tumor cells (DTCs) in the bone marrow (BM), and we characterized CTCs for the expression of HER2, the estrogen receptor (ER) and the progesterone receptor (PR).

**Methods:**

Blood of 431 patients with primary breast cancer were analyzed for EpCAM, MUC1 and HER2 transcripts with the *AdnaTest BreastCancer*™ (AdnaGen AG, Germany). Expression of the ER and PR was assessed in an additional RT-PCR. BM aspirates from 414 patients were analyzed for DTCs by immunocytochemistry using the pan-cytokeratin antibody A45-B/B3.

**Results:**

DTCs were found in 107/414 patients (24%), CTCs were detected in 58/431 (13%) patients. DTCs were associated with PR status of the primary tumor (*P *= 0.04) and CTCs significantly correlated with nodal status (*P *= 0.04), ER (*P *= 0.05), and PR (*P *= 0.01). DTCs in the BM weakly correlated with CTCs (*P *= 0.05) in blood. Interestingly, the spread of CTCs was mostly found in triple-negative tumors (*P *= 0.01) and CTCs in general were mostly found to be triple-negative regardless of the ER, PR and HER2 status of the primary tumor.

**Conclusions:**

(1) Due to the weak concordance between CTCs and DTCs the clinical relevance may be different. (2) The biology of the primary tumor seems to direct the spread of CTCs. (3) Since the expression profile between CTCs and the primary tumor differs, the consequence for the selection of adjuvant treatment has to be evaluated.

## Introduction

Disseminated tumor cells (DTCs) in the bone marrow (BM) are a common phenomenon seen in breast cancer at primary diagnosis in up to 40% of patients [1-3]. Tumor cell detection in the BM is regarded increasingly as a clinically relevant prognostic factor for breast cancer, and a pooled analysis of BM findings in more than 4,700 patients documented that their presence is associated with a poor prognosis [4]. In addition, it has been demonstrated that tumor cells frequently survive chemotherapy [5] and the persistence of these cells in BM after conventional adjuvant chemotherapy is associated with poor prognosis [6-8]. The consequences for a possible secondary adjuvant therapy are under discussion. Since BM aspiration is less accepted by patients compared with blood drawing, it would be highly desirable to replace BM aspiration by blood analysis.

For the detection of circulating tumor cells (CTCs) in blood, both antibody-based assays using antibodies against epithelial specific markers (for example, cytokeratins (CKs)) and molecular assays (for example, based on amplification of epithelial-specific mRNA transcripts) have been established [9-20].

The most currently used approach, particularly in clinical trials, is the CellSearch™ System (Veridex, Warren, NJ, USA), which is as a semi-automated device based on immunofluorescence and flow cytometry. CTCs are isolated by immunomagnetic beads coated with antibodies against the epithelial cell adhesion molecule (EpCAM) and are identified by CK positivity, positive nuclear staining and CD45 negativity [9,10,18].

Another commercially available CTC detection system is the *AdnaTest BreastCancer*™ (AdnaGen AG, Langenhagen, Germany). First, CTCs are isolated by immunomagnetic beads labeled with antibodies against MUC1 and EpCAM. After isolation of the mRNA, transcripts of epithelial-specific markers (GA 73.3, EpCAM and human epidermal growth factor receptor 2 (HER2)) are amplified by a multiplex PCR [21-23].

Based on current data, the concordance rate of the CellSearch™ assay and the *AdnaTest BreastCancer*™ ranges between 68% and 88% [24,25]. A prospective multicenter trial in metastatic breast cancer using both methods – the CellSearch™ assay and the *AdnaTest BreastCancer*™ – to re-evaluate the HER2 status by circulating CTC has recently completed its recruitment, but the data on concordance have not yet been published [26].

The clinical significance of CTCs in metastatic breast cancer has been clearly demonstrated [9,10]. In contrast, the predictive and prognostic value of CTCs in primary breast cancer is still under investigation [11-17]. Single-center studies have indicated that the presence of CTCs at the time of first diagnosis is an independent prognostic factor for overall and disease-free survival [11-13,27,28]. In addition, patients with CTC persistence after completion of systemic cytotoxic therapy are more likely to develop relapse compared with those who were CTC-negative [29,30]. Targeting persistent CTCs is therefore an important issue to improve prognosis in primary breast cancer patients.

In this context, the need to determine the expression of therapeutic relevant markers in minimal residual disease is becoming increasingly important to optimize adjuvant treatment. Adjuvant therapy targets minimal residual disease, which is reflected by CTCs and DTCs. It has already been shown that the expression of therapeutic relevant markers including estrogen receptor (ER) and HER2 may differ from that of the primary tumor [31,32]. These observed discrepancies may be the cause of tumor cell persistence and therapeutic failure in the adjuvant setting.

In the present study we have used a RT-PCR-based approach to examine the presence of CTCs in peripheral blood of patients with primary breast cancer, to assess the correlation between CTCs in blood and DTCs in BM, and to compare the expression profile of therapeutic relevant markers (HER2, ER, progesterone receptor (PR)) between CTCs and the primary tumor.

## Materials and methods

### Patients' characteristics

The study was conducted at the Department of Obstetrics and Gynecology in Essen and in the Department of Obstetrics and Gynecology in Tübingen. In total, 431 primary breast cancer patients (pT1 to pT4, pN0 to PN2, Mo) have been studied. Patients' characteristics at the time of diagnosis are presented in Table 1.

**Table 1:** Clinical data of patients

	Total	CTC-positive	*P *value	Total	DTC-positive	*P *value
Total	431	58 (13)	414	107 (24)		
Tumor size						
pT1	285	34 (12)	0.47	274	66 (24)	0.07
pT2 to pT4*	134	21 (16)	130	40 (31)		
bilateral	10	2 (20)	9	0 (0)		
Nodal status						
Node-negative	286	32 (11)	0.04	273	71 (26)	0.92
Node-positive	142	26 (18)	137	35 (26)		
Histology						
Ductal	349	45 (13)	0.33	337	85 (25)	0.54
Lobular	65	12 (19)	60	16 (27)		
Others	16	1 (6)	16	6 (38)		
Grading						
I	58	6 (10)	0.11	57	14 (25)	0.76
II	270	32 (12)	255	64 (25)		
III	102	20 (20)	101	29 (29)		
ER status						
Negative	60	13 (22)	0.05	57	15 (26)	0.93
Positive	371	45 (12)	357	92 (26)		
PR status						
Negative	75	17 (23)	0.01	70	25 (36)	0.04
Positive	356	41 (12)	344	82 (24)		
HER2						
Negative	380	49 (13)	0.35	365	89 (24)	0.06
Positive	51	9 (18)	49	18 (37)		
Bone marrow status						
Positive	106	20 (19)	0.05			
Negative	299	34 (11)				
Immunhistochemical subtype						
(ER^-^, PR^-^, HER2^-^)	26	8 (30)	0.01	23	7 (30)	0.62
(ER^-^, PR^-^, HER2^+^)	11	0	11	4 (36)		
(ER^+ ^and/or PR^+^)	394	50 (13)	380	96 (25)		

ER, estrogen receptor; HER2, human epidermal growth factor receptor 2; PR, progesterone receptor.

### Collection and analysis of bone marrow

Between 10 and 20 ml BM were aspirated from the anterior iliac crests of 414 out of 431 primary breast cancer patients at the time of surgery and were processed within 24 hours. All specimens were obtained after written informed consent and were collected using protocols approved by the institutional review board (Protocol 114/2006A and Protocol 05/2856). Tumor cell isolation and detection was performed based on the recommendations for standardized tumor cell detection recently published by the German Consensus Group of Senology [33].

BM cells were isolated from heparinized BM (5,000 U/ml BM) by Ficoll-Hypaque density gradient centrifugation (density 1.077 g/mol; Pharmacia, Freiburg, Germany) at 400 × *g *for 30 minutes. Interface cells were washed (400 × *g *for 15 min) and resuspended in PBS. Then 1 × 10^6^mononuclear cells per area of 240 mm^2 ^from each aspiration side were directly spun onto glass slides (400 × *g *for 5 min) coated with poly-l-lysine (Sigma, Deisenhofen, Germany) using a Hettich cytocentrifuge (Tuttlingen, Germany) for the detection of CK-positive cells. The slides were air-dried overnight at room temperature.

### Immunocytochemistry

Staining for CK-positive cells was performed using the murine mAb A45-B/B3 (Micromet, Munich, Germany), directed against a common epitope of CK polypeptides including the CK heterodimers 8/18 and 8/19 [13,27,28,32]. The protocol has been described in detail elsewhere [34]. Briefly, the method includes permeabilization of the cells with a detergent (5 min), fixation with a formaldehyde-based solution (10 min), binding of the conjugate mAb A45-B/B3-alkaline phosphatase to cytoskeletal CKs (45 min), and formation of an insoluble red reaction product at the site of binding of the specific conjugate (15 min) using the DAKO-APAAP detection kit (DakoCytomation, Glostrup, Denmark) according to the manufacturer's instructions. Subsequently, the cells were mounted with Kaiser's glycerol/gelatine (Merck, Darmstadt, Germany) in Tris-ethylenediamine tetraacetic acid buffer (Sigma). A control antibody (conjugate of Fab fragment; Micromet) served as negative control. For each test a positive control slide with the breast carcinoma cell line MCF-7 (ATCC, Rockville, MD, USA) was treated under the same conditions.

### Evaluation of CK-positive cells

Microscopic evaluation of the slides was carried out using the ACIS system (Chromavision, San Juan Capistrano, CA, USA) at the Department of Gynecology and Obstetrics, Tübingen and using the ARIOL system (Applied Imaging, Newcastle upon Tyne, UK) at the Department of Gynecology and Obstetrics, Essen according to the ISHAGE evaluation criteria and the DTC consensus [19,20]. These automated scanning microscopes and image analysis systems consist of a slide loader, camera, computer and software for the detection and classification of cells of interest based on particular color, intensity, size, pattern, and shape. All slides were evaluated at both the Tübingen (TF) and Essen (SK-B) centers. In case nonconcordant results were obtained, slides were evaluated by a third investigator (EFS) to obtain consensus.

### Sampling of blood

Two samples of 5 ml ethylenediamine tetraacetic acid blood were collected for isolation of CTCs before the application of therapeutic substances with an S-Monovette^® ^(Sarstedt AG & Co, Nümbrecht, Germany) and were stored at 4°C until further examination. The samples were processed immediately or not later than 4 hours after blood withdrawal. An additional serum sample was collected to determine serum tumor markers.

### Tumor cell enrichment/selection

Blood samples were taken from 431 patients and were analyzed for CTCs with the *AdnaTest BreastCancer*™ (AdnaGen AG), which enables the immunomagnetic enrichment of tumor cells via epithelial and tumor-associated antigens. Establishment and validation of the *AdnaTest BreastCancer*™ assay has been described in detail elsewhere [21-23,35]. The lower detection limit of this assay is based on spiking experiments two cells per 5 ml [35]. Examination of blood samples of healthy donors (n = 106) yielded a specificity of 95% [35].

In brief, blood samples were incubated with a ready-to-use antibody mixture (against GA 73.3 and MUC1) commercialized as *AdnaTest BreastCancerSelect *(AdnaGen AG, Langenhagen, Germany) according to the manufacturer's instructions. The labeled cells were extracted by a magnetic particle concentrator. Subsequently, mRNA isolation from lysed, enriched cells was performed according to the manufacturer's instructions with the Dynabeads mRNA DIRECT™ Micro Kit (Dynal Biotech GmbH, Hamburg, Germany) that is included in the *AdnaTest BreastCancerDetect *(AdnaGen AG). Reverse transcription resulted in cDNA, which was the template for tumor cell detection and characterization by multiplex RT-PCR. Sensiscript^® ^Reverse Transcriptase (QIAGEN GmbH, Hilden, Germany) was used for the reverse transcription because of its high sensitivity (recommended for < 50 ng RNA) in combination with oligo(dT)-coupled Dynabeads of the mRNA DIRECT™ Micro Kit (Dynal Biotech GmbH, Hamburg, Germany) according to the manufacturer's instructions [35].

### Tumor cell detection

The *Adnatest BreastCancerDetect *was used for the detection of breast cancer-associated gene expression in immunomagnetically enriched tumor cells by reverse transcription and PCR. The analysis of tumor-associated mRNA isolated from CTCs was performed in a multiplex PCR for the three tumor-associated transcripts HER2, MUC1 and GA 733-2 followed by storage of the samples at 4°C.

The primer sets for the ER and PR receptor were provided by Adnagen AG. These reagents detected ER and PR on CTCs after the preparation of the cDNA and according to the manufacturer's instructions of the *AdnaTest BreastCancerDetect*. PCR was performed with the HotStarTaq Master Mix (QIAGEN GmbH). Actin was used as the internal PCR positive control. The thermal profile used for the nested RT-PCR was as follows. After a 15-minute denaturation at 95°C, 37 cycles of PCR were carried out by denaturation at 94°C for 30 seconds, annealing/extension at 60°C for 30 seconds and elongation for 30 seconds at 72°C. Termination of the reaction was subsequently carried out at 72°C for 5 minutes followed by storage of the samples at 4°C.

The primers generate fragments of the following sizes: GA 733-2, 395 bp; MUC1, 293 bp; HER2, 270 bp; PR, 270 bp; ER, 305 bp; and actin, 114 bp. Visualization of the PCR fragments was carried out with a 2100 Bioanalyzer using the DNA 1000 LabChips (Agilent Technologies, Santa Clara, CA, USA) and the Expert Software Package (Agilent Technologies; version B.02.03.SI307).

### Evaluation of data established for circulating tumor cells

The test is considered positive if a PCR fragment of at least one tumor-associated transcript (MUC-1, GA 733-2 or HER2) is clearly detected. Using the software package for evaluation of the data on the Agilent 2100 Bioanalyzer, peaks with a concentration > 0.15 ng/μl are positive for the transcripts GA 733-2, MUC1 and HER2. Peaks with a concentration > 0.60 ng/μl are positive for the ER transcript. The PR expression is considered positive when the transcript is detected without applying any cutoff value.

### Immunohistochemical analysis of the primary tumor

For each of the 431 patients, the tumor type, TNM staging and grading were assessed according to the World Health Organization Classification of tumors of the breast [36] and the sixth edition of the TNM Classification System [37].

The ER status and PR status were determined by immunohistochemistry. Sections of 5 μm thickness were cut and mounted on SuperFrost^® ^Plus slides (Menzel, Braunschweig, Germany). Following individually optimized heat-based antigen retrieval for each antibody, each glass slide was immunostained with commercially available antibodies. The following antibodies were used: anti-ER (clone SP1; DCS, Hamburg, Germany), dilution 1:300, antigen retrieval for 30 minutes in a 95°C waterbath, citrate buffer, pH 6.0; and anti-PR (clone 16; DCS), dilution 1:200, antigen retrieval for 30 minutes in a 95°C waterbath, citrate buffer, pH 6.0.

Automated immunohistochemistry was performed using the Dako Autostainer Plus System (DakoCytomation, Carpinteria, CA, USA) with the anti-mouse IgG EnVision Plus detection kit (DakoCytomation) for secondary and tertiary immunoreactions. Reaction products were developed with diaminobenzidine, according to general protocols. Positive and negative control sections were included in each run, which showed appropriate results.

The DAKO score for the expression of HER2 was determined with the HercepTest, and fluorescence *in situ *hybridization analysis in cases of 2+ staining as determined with the HercepTest was performed as described elsewhere [38].

### Statistical analysis

The chi-squared test or Fisher's exact test was used to evaluate the relationship between DTCs and CTCs and clinicopathological factors. Statistical analysis was performed with SPSS software (version 11.5; SPSS Inc., Chicago, IL, USA). *P *< 0.05 was considered statistically significant.

## Results

### Patients' characteristics

A total of 431 patients were included in the study: 285 out of 431 (66%) patients had T1 tumors, and 286 out of 431 (66%) patients were node-negative. The expression of therapeutic relevant markers in primary tumors was available from all 431 patients. ER and PR positivity was observed in 86% (371/431) and 83% (356/431) of the tumors, respectively. HER2 was overexpressed in 12% (51/431) of the cases. Classifying tumors into subtypes based on their ER, PR and HER2 expression, 91% of the tumors were ER-positive and/or PR-positive, 6% were triple-negative (ER-negative/PR-negative/HER2-negative) and 11 tumors only expressed HER2 (ER-negative/PR-negative/HER2-positive). Clinical data are presented in detail in Table 1.

### Incidence of circulating tumor cells in blood

A blood sample was regarded CTC-positive if at least one of the three markers GA 733-2, MUC1 or HER2 was expressed. The detection rate for CTCs was 13% (58/431 patients) with the expression rates of 55% for EpCAM (32/58 patients), 53% for MUC1 (31/58 patients) and 38% for HER2 (22/58 patients) (Table 2). The presence of CTCs significantly correlated with positive nodal status (*P *= 0.04), negative ER (*P *= 0.05) and negative PR (*P *= 0.01), respectively. The highest CTC positivity rate was obtained in triple-negative patients followed by those with ER-positive and/or PR-positive tumors (30% vs. 13%, *P *= 0.01). No CTCs could be detected in the HER2-positive subtype group (Table 1).

**Table 2:** Expression profile of circulating tumor cells in breast cancer patients compared with the primary tumor

		Circulating tumor cells		Primary tumor (n = 58)		Concordance (%)
			
	*n*	*n*	%	*n*	%	
ER-positive	48	12	25	45	78	29
PR-positive	48	2	2	41	71	25
HER2-positive	58	22	38	9	16	53
Muc-1-positive	58	31	53	-	-	-
GA 73.3-positive	58	32	55	-	-	-

ER, estrogen receptor; HER2, human epidermal growth factor receptor 2; PR, progesterone receptor.

### Correlation of disseminated and circulating tumor cells with established prognostic markers

The BM status of DTCs was available in 414 patients. DTCs were found in 107 out of 414 (24%) patients. No correlation could be observed between positive BM status and the established prognostic markers except for the PR status of the primary tumor (*P *= 0.04; Table 1). The CTC status was weakly associated with the presence of DTCs in BM (*P *= 0.05; Table 1). The positivity rate was 19% in patients with positive BM status compared with 11% in those with negative BM status.

### Expression profiling of circulating tumor cells and corresponding tumors

**Figure 1:**
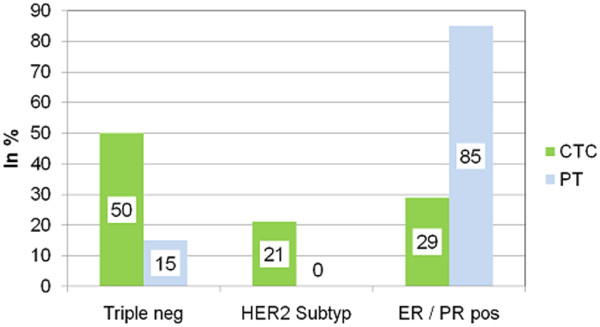
Expression of circulating tumor cells and corresponding primary tumors based on receptor status. Expression profile of circulating tumor cells (CTCs) and corresponding primary tumors (PTs) in breast cancer patients after stratifying into three different groups based on estrogen receptor (ER), progesterone receptor (PR) and human epidermal growth factor receptor 2(HER2) status: ER-positive and/or PR-positive, triple-negative (ER-negative/PR-negative/HER2-negative), and HER2-positive (but ER-negative/PR-negative).

The expression profile of CTCs included ER, PR, and HER2. The expression rates were 38% for HER2 (22/58 patients), 25% for ER (12/48 patients) and 4% for PR (2/48 patients). In 10 patients, the ER and PR expression could not be determined in CTCs due to the small sample volume. Regarding the immunohistochemical subtype, 50% of the CTCs (24/48 patients) were triple-negative and 21% (10/48 patients) were only HER2-positive. The remaining 29% of CTCs (14/48 patients) were ER-positive and/or PR-positive with positive or negative HER2 status. Comparing the expression profile between CTCs and the primary tumor, ER positivity of the primary tumor was demonstrated in 45 out of 58 (78%) of these patients, PR positivity in 41 out of 58 (71%) patients and HER2 positivity in 9 out of 58 (16%) patients. The concordance rate between ER, PR and HER2 status of CTCs and the primary tumor was 29%, 25% and 53%, respectively (Table 2). Interestingly, CTCs were triple-negative in 50% (24/48) of all cases whereas only 15% (7/48) of the primary tumors were negative for ER, PR and HER2 (see Figure 1).

## Discussion

Recent publications have indicated that the presence of CTCs and DTCs in peripheral blood and BM, respectively, does not provide congruent results and that there is a more complex relationship between the primary tumor and DTCs on the phenotype level as well on the genomic level. We demonstrate here, in a large patient group: a weak correlation between the presence of CTCs in peripheral blood and the presence of DTCs in BM; that CTCs are more closely related to the biology of the primary tumor than DTCs; that the majority of CTCs are triple-negative, regardless of the antigen expression of the primary tumor; and, most interestingly, that CTCs mostly derive from triple-negative tumors.

### Incidence of circulating tumor cells in primary breast cancer

Using a multiplex RT-PCR approach, our positivity rate was 13% in primary breast cancer. Detection rates reported by other researchers using different approaches range from 9% to 50% depending on the clinical stages included [11-21,27-30]. There is still currently an ongoing debate whether the (RT-)PCR-based approach (for example, the *AdnaTestBreastCancer*™) or the antibody-based approach (for example, the CellSearch™ system) is superior in a clinical setting to monitor therapy response and to predict prognosis.

False positive results can be obtained using both the molecular approach and the antibody-based assay since only epithelial but not unique breast cancer specific markers are available for identification of breast cancer tumor cells. These markers can also be expressed in contaminating stimulated leukocytes [19,39]. The antibody-based approach allows a morphological assessment to confirm malignancy based on cytomorphological criteria [33,34]. In contrast, the RT-PCR approach seems to be more sensitive and allows easy multiple marker testing [39,40].

Until now, only few studies have compared both techniques [40,41]. Trials have been initiated (for example, the DETECT Study) to determine the CTC status using both the antibody-based assay and the molecular-based approach to elucidate which assay provides more valid data in predicting therapy response and prognosis [26].

### Correlation between circulating tumor cells in peripheral blood and disseminated tumor cells in bone marrow

Some research groups compared the relationship between the presence of DTCs in BM and CTCs in blood [15-18]. In general, correlation between DTCs and CTCs is low. It has been demonstrated that, in contrast to the detection of DTCs in BM of patients with early disease, detection of CTCs appears to be less sensitive and less prognostic [16,17]. Furthermore, there is growing evidence that the prognosis of women with primary breast cancer depends on DTCs rather than CTCs [15,16,42]. In our study, there was a weak correlation between the presence of CTCs in peripheral blood and the presence of DTCs in BM, and the presence of CTCs was more closely related to the biology of the primary tumor than DTCs. Only the clinical follow-up data of the patients, however, will elucidate the prognostic significance of either CTCs or DTCs, or both.

### Expression profile of circulating tumor cells compared with the primary tumor

The aim of adjuvant treatment is to eliminate minimal residual disease reflected by CTCs and DTCs. The predictive markers ER, PR and HER2, however, are determined in the primary tumor to determine whether HER2 targeted therapy and endocrine therapy will be effective. One of the main objectives of the present paper was therefore to determine the concordance of the predictive markers ER, PR and HER2 between CTCs and corresponding primary tumors.

On the phenotype level, the expression of HER2 on DTCs or CTCs of patients with primary breast cancer has been published by different groups – who independently indicated that HER2 expression on both DTCs and CTCs differed from HER2 expression in the primary tumor and that the expression of HER2 on DTCs and CTCs was correlated with poor prognosis [32,43-46]. These observations might have clinical relevance when selecting patients for HER2 targeted therapy. Patients with HER2-negative tumors but HER2-positive CTCs might also benefit from HER2 targeted therapy. In a metastatic setting, Meng and colleagues have already shown that metastatic patients who were regarded as HER2-negative on the basis of HER2 expression of their primary tumor had circulating HER2-positive cells and responded to trastuzumab [47].

Similarly, the hormonal status of DTCs and CTCs could be completely different from that of the primary tumor, which on the one hand (tumor-negative, DTC/CTC-positive) could increase the number of patients eligible for endocrine therapy and on the other (tumor-positive, DTC/CTC-negative) could explain why endocrine therapy fails in a subset of hormone receptor-positive patients. Ditsch and colleagues, in an observational study looking at 17 primary tumors and their corresponding DTCs, found that only 2 out of 11 patients (18%) with ERα-positive primary tumors had ERα-positive DTCs [48]. We recently demonstrated in a cohort of 254 patients with primary breast cancer that the primary tumor and DTCs in BM displayed a concordant ERα status in only 28% of cases [31].

Studies comparing the expression of HER2 as well as ER/PR expression of the primary tumor with CTCs have rarely been published. As already published in our study on metastatic breast cancer, we here show that CTCs were more likely to be ER-negative and PR-negative compared with the primary tumor. In contrast, CTCs were HER2-positive in 38% of the patients whereas only 16% of the corresponding primary tumors expressed HER2 [49,50]. The results are therefore similar to the data derived from the DTC studies.

These phenotypical changes or discrepancies between the primary tumor, DTCs and CTCs are not confined to hormonal receptors and HER2 alone, since other studies looking at markers such as MHC III, Ki-76 and EGF-R have reported similar discrepancies [51-53]. In summary, all published data suggest that CTCs and DTCs may represent a unique and heterogeneous cell population.

Looking at a large patient group, our data confirm these previous findings of smaller studies, indicating that reliance on the phenotype of the primary tumor can be misleading since HER2 as well as the hormonal receptors were differently expressed on CTCs as compared with the primary tumor. These results have major implications for selecting adjuvant treatment. Since the expression profile of predictive markers of DTCs and CTCs, the target of adjuvant treatment, differs from the corresponding primary tumor, the next step is to design a clinical trial stratifying adjuvant treatment based on the expression profile of CTCs or DTCs versus the primary tumor to determine whether patients will derive more benefit from therapy selected based on the expression profile of minimal residual disease.

### Characterization of circulating tumor cells and corresponding shedding primary tumors

On stratifying our patients into three different groups according to the tumor subtype classifications, the majority of patients had ER/PR-positive tumors. Only 6% of the patients were triple-negative, which is low compared with reported incidences of 15 to 30% [54]. The low incidence may be due to the fact that nearly all patients included in this study were Caucasian and had a high socioeconomic status (for example, not obese) [54,55].

CTCs were mostly found in patients with triple-negative tumors. In addition, most of the CTCs were triple-negative regardless of the subtype of the primary tumor. Different hypotheses need to be discussed with regard to our findings. One possible explanation is the clonal heterogeneity of the primary tumor, allowing only a certain subpopulation to disseminate. On first view, it seems that ER-negative, PR-negative and HER2-negative cells could more probably disseminate, corresponding to the worse prognosis of predominantly ERα-negative tumors and – inversely – to the demonstrated decreased invasiveness and metastatic potential of ERα-expressing breast cancer cells [56,57].

One currently discussed hypothesis is the theory that some DTCs or CTCs, the presumed precursor cells of systemic metastatic disease, are in fact cancer stem cells. As recently published, this theory states that tumor growth and formation of secondary tumors can be traced to a small subpopulation of tumor cells, so-called cancer stem cells [58,59]. At least one study has confirmed a putative stem cell phenotype in DTCs [60], and another study has shown that the majority of early DTCs detected in the BM of breast cancer patients with a CD44^+^/CD24^- ^phenotype correlated with a higher prevalence of bone metastases [61]. As breast cancer stem cells have been shown to be generally triple-negative, triple-negative CTCs are in concordance with the cancer stem cell theory [62,63].

## Conclusions

Two major conclusions can be drawn from these results. First, CTCs and DTCs have different meanings due to the low concordance. To determine the difference in predicting prognosis, clinical follow-up is needed. Second, CTCs have a different expression profile compared with the primary tumor. The impact on adjuvant treatment can only be answered in clinical trials randomizing patients according to the expression profile based on CTCs or DTCs.

## Abbreviations

BM: bone marrow; bp: base pairs; CK: cytokeratin; CTC: circulating tumor cell; DTC: disseminated tumor cell; EpCAM: epithelial cell adhesion molecule; ER: estrogen receptor; HER2: human epidermal growth factor receptor 2; mAb: monoclonal antibody; PBS: phosphate-buffered saline; PCR: polymerase chain reaction; PR: progesterone receptor; RT: reverse transcriptase.

## Competing interests

The authors declare that they have no competing interests.

## Authors' contributions

TF, BA, OH, SV and SK-B made substantial contributions to the conception and design of the study, acquisition of data, and analysis and interpretation of the data. TF, SK-B, EFS, DW and RK were involved in drafting the manuscript or revising it. All authors read and approved the final manuscript.
